# Causes and predictors of recurrent unplanned hospital admissions in heart failure patients: a cohort study

**DOI:** 10.1007/s11739-024-03740-2

**Published:** 2024-08-18

**Authors:** Ofra Kalter-Leibovici, Havi Murad, Arnona Ziv, Tomer Keidan, Alon Orion, Yoav Afel, Harel Gilutz, Dov Freimark, Rachel Klibansky-Marom, Laurence Freedman, Haim Silber

**Affiliations:** 1 The Gertner Institute for Epidemiology and Health Policy Research, Sheba Medical Center, Ramat-Gan, Israel; 2 School of Public Health, Faculty of Medical & Health Sciences, Tel-Aviv University, Tel-Aviv, Israel; 3 Department of Surgery, UF Health, University of Florida, Gainesville, USA; 4 The Edmond and Lily Safra Children’s Hospital, Sheba Medical Center, Ramat-Gan, Israel; 5 Olga and Lev Leviev Heart Center, Sheba Medical Center, Ramat-Gan, Israel; 6 Clalit Research Institute, Tel-Aviv, Israel; 7 Heart Institute, Marom Medical Center, Kfar-Saba, Israel

**Keywords:** Chronic heart failure, Unplanned hospital admissions, Cluster analysis, Risk factors, Prediction

## Abstract

Despite progress in therapy, heart failure (HF) inflicts a heavy burden of hospital admissions. In this study, we identified among 1360 community-dwelling HF patients (mean age 70.7 ± 11.3 years, 72.5% men) subgroups sharing similar profiles of unplanned hospital admissions, based on the admission causes and frequency of each cause. Hospital discharge summaries were reviewed for the main admission cause. Patient subgroups were identified via cluster analysis. We investigated baseline predictors associated with these subgroups, using multinomial logistic models. During 3421 patient-years, there were 5192 hospital admissions, of which 4252 (82%) were unplanned. We identified five patient subgroups (clusters 1–5) with distinctive hospitalization profiles. HF accounted for approximately one-third of admissions in the first patient cluster (23% of the patient sample). In contrast, patients in the second cluster (39% of the patient sample) were hospitalized for various reasons, with no single prominent admission cause identified. The other three clusters, comprising 16% of the patient sample, accounted for 42% of all unplanned hospitalizations. While patients in the third cluster were hospitalized mainly due to ischemic heart disease and arrhythmia, patients in the fourth and fifth clusters shared a high burden of recurrent HF admissions. The five patient clusters differed by baseline predictors, including age, functional capacity, comorbidity burden, hemoglobin, and cause of HF. HF patients differ significantly in the causes and overall burden of unplanned hospitalizations. The patient subgroups identified and predictors for these subgroups may guide personalized interventions to reduce the burden of unplanned hospitalizations among HF patients. *Trial registration:* ClinicalTrials.gov, NCT00533013. Registered 20 September 2007. https://clinicaltrials.gov/study/NCT00533013.

**Supplementary Information:**

The online version contains supplementary material available at 10.1007/s11739-024-03740-2.

## Introduction

Despite significant improvements in management, heart failure (HF) is associated with high mortality rates [1, 2], poor functional capacity, and impaired quality of life [3]. HF also inflicts a heavy burden on healthcare systems, mostly because of recurrent hospital admissions [4–7]. Approximately 20–25% of patients hospitalized for acute HF in the United Kingdom [6] and the United States [7] were readmitted to the hospital within 30 days. Nevertheless, HF was the primary diagnosis for only 28–35% of these readmissions [6, 7]. While hospital readmissions for HF and other cardiovascular diseases remained stable between 2002 and 2018 in the United Kingdom, hospitalizations for non-cardiovascular causes increased by 2.6% per year [8]. In the United States, non-cardiovascular causes account for more than half of hospital admissions among HF patients [1, 9]. A recent study in Denmark showed that the proportion of hospital admissions for non-HF causes was even higher in the last year of life among HF patients, with other cardiovascular causes (e.g., arrhythmia, ischemic heart disease, cerebrovascular disease) and non-cardiovascular morbidity, accounting for approximately 18% and 64% of all admissions, respectively [10].

Disease management programs, which focus mainly on improving HF management and patient adherence to the treatment plan, have varying efficacy in reducing unplanned hospital admissions among HF patients. The interventions tested were commonly multifactorial, including patient education, telephone calls and home visits, tele-monitoring, and multidisciplinary care [11–15]. Since a significant proportion of hospital admissions among HF patients are not due to acute exacerbation of HF, interventions focused only on HF management may have limited efficacy in reducing the overall burden of unplanned hospitalizations. Furthermore, the effectiveness of such multifactorial interventions may vary by patient characteristics and by causes and frequency of hospital admissions.

In this study, we aimed to identify subgroups of patients with chronic HF sharing a similar profile of unplanned hospital admissions by cause and frequency and to study baseline predictors of these subgroups.

## Methods

This is an in-depth analysis of information collected in a randomized controlled trial. The trial tested the efficacy of a multifactorial disease-management intervention in community-dwelling patients with chronic HF. The disease management intervention tested was not more effective than usual care in reducing hospital admissions or all-cause mortality [16].

The study sample and procedures have been previously described [16]. In brief, 1360 ambulatory adult patients (age ≥ 18 years) who were insured by Maccabi Health Services, the second largest health plan in Israel and diagnosed with moderate-to-severe chronic heart failure [New York Heart Association (NYHA) functional class II to IV] were included in the study. Patients were referred for eligibility screening after recent hospital admission for heart failure exacerbation (38%) or from the community (62%). Patients were included regardless of their heart failure etiology or left ventricular ejection fraction (LVEF). Patients who were bedridden or burdened with a terminal disease were excluded.

The patients were recruited between August 2007 and June 2011 and assessed at recruitment and every six months afterward until death or the end of the study (July 2012). The baseline information collected included demographic, clinical, and functional capacity data. Information on hospital admissions or deaths that occurred during follow-up was collected from administrative databases. The median follow-up period was 2.6 years (range 0–5). The study was approved by the Maccabi Health Services and the Sheba Medical Centre research ethics committees (approval ID numbers 2007045 and 4807/07, respectively). All patients provided written informed consent before inclusion. The original trial protocol was registered at Clinicaltrails.gov (identifier: NCT00533013).

For the current analysis, trained physician assessors, masked to the patient identifying information and assigned intervention, reviewed all discharge summaries of hospital admissions of more than 1-day duration occurring during follow-up. One-day hospitalizations were included only in cases of in-hospital death on the day of admission. Eligible hospital admissions were classified as planned or unplanned, and the main admission cause was coded using the ninth revision of the International Classification of Diseases (ICD-9).

The main causes of unplanned hospital admissions were grouped into 43 diagnosis categories according to body system/disease type and frequency (Table 1 in Supplementary Information 1). Almost half of the discharge summaries (*N* = 1980; 47%) were analyzed by two or more assessors (up to five assessors per single discharge summary). The between-observer agreement on the diagnostic category of the main admission cause was good; weighted Kappa = 0.84. Disagreements between assessors were discussed and resolved by the investigators.

**Table 1:** Patient subgroups with similar unplanned hospital admission profile, by cause and frequency: results of cluster analysis*

Main cause of hospital admission	Mean number of hospital admissions/person during follow-up				
	Cluster 1 *N* = 307	Cluster 2 *N* = 528	Cluster 3 *N* = 49	Cluster 4 *N* = 115	Cluster 5 *N* = 56
Heart failure	1.3	0.2	0.9	3.6	7.8
Coronary artery disease	0.2	0.2	2.0	0.2	0.7
Arrhythmia/conduction disturbance	0.1	0.2	2.1	0.2	0.4
Acute bronchitis/pneumonia	0.4	0.1	0.2	0.2	0.4
Obstructive/restrictive lung disease	0.1	0.1	0.0	0.0	0.4
Anemia	0.0	0.1	0.1	0.3	0.1
Mean (standard deviation) number of total unplanned hospital admissions per patient during follow-up	**3.5 (2.6)**	**2.7 (2.0)**	**6.9 (3.3)**	**6.6 (2.8)**	**12.1 (8.4)**

The numbers in bold reflect the mean number and standard deviation of unplanned hospital admissions from all causes during follow-up, as explained in the left column

*The table includes a selected list of main admission causes that were more frequent in at least one patient cluster. Data on the mean admission number for the complete list of diagnosis groups (*N* = 43) are presented in Supplementary Information 1

### Statistical analysis

For each patient with at least one unplanned hospital admission, a profile of the number of unplanned hospital admissions due to each main cause was built. We ran a cluster analysis to identify homogenous and distinct subgroups of patients who shared a similar profile of recurrent hospital admissions (according to the main admission causes and the frequency of each cause). First, a Euclidean distance matrix was calculated between each pair of subjects based on these profiles. For example, for two identical profiles, the distance was zero and increased as the profiles diverged. We then conducted a hierarchical cluster analysis [17] based on the distance matrix and the Ward criterion [18]. We also checked the clinical interpretation of the final cluster solution.

A separate subgroup was created for patients who did not experience an unplanned hospital admission during follow-up.

Univariate comparisons of baseline demographic and clinical characteristics by patient subgroups (i.e., study outcome) were made with the Kruskal‒Wallis H and chi-square statistics for continuous/ordered variables and contingency tables, respectively. Post hoc comparisons between patient subgroups, i.e., patients who had no unplanned hospital admissions and the patient clusters identified, were tested with the Wilcoxon test.

We used multinomial logistic regression models (with reference category comprising patients with no unplanned hospital admission during follow-up) to test which baseline characteristics were independently associated with the patient clusters identified. Variables were entered into the model if associated with cluster membership with a *p*-value < 0.2 in univariate analysis. Backward variable selection, with a *p*-value < 0.1, was used to select variables for the final model. All models were adjusted for patient age, sex, assigned intervention (i.e., disease management or usual care), and length of follow-up.

The goodness of prediction of the multinomial model was checked using a series of algorithms for assigning patients to their subgroups (patients who did not experience an unplanned admission and clusters 1–5) based on the estimated probability derived from the model to belong to each subgroup.

We evaluated the goodness of prediction provided by each assignment algorithm using classification tables in conjunction with ascribed losses corresponding to each type of misclassification. The assignment algorithm that yielded the lowest total loss was considered optimal (Supplementary Information 3).

## Results

**Fig. 1:**
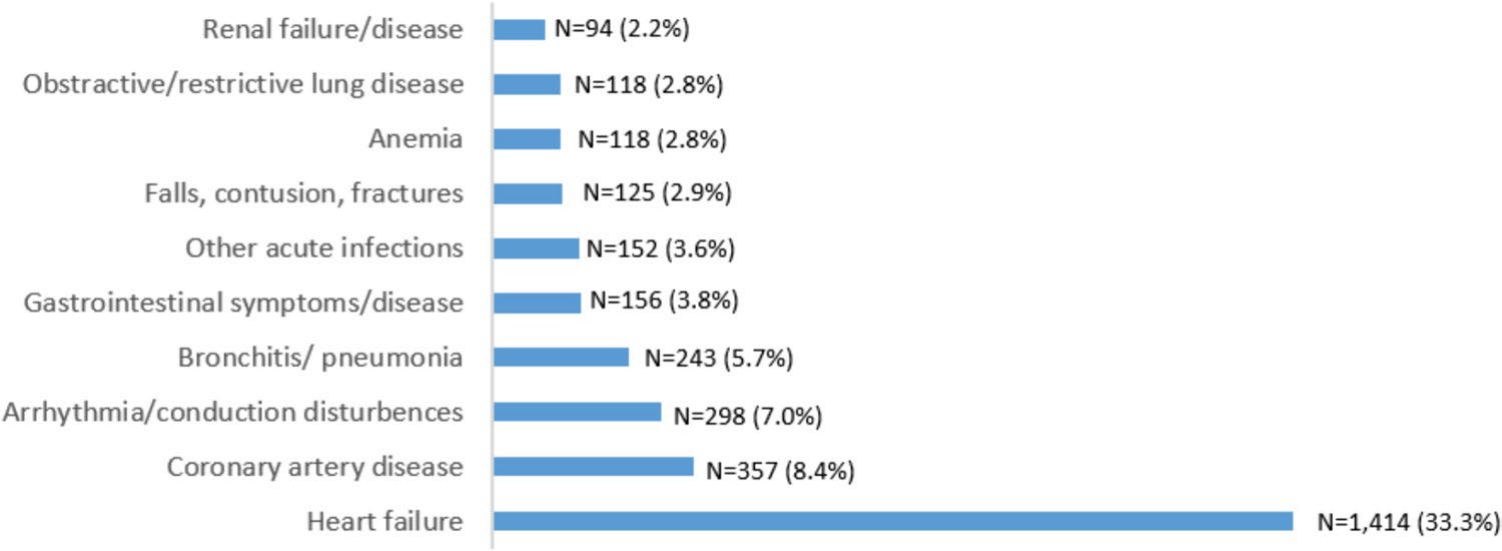
The ten most frequent causes for unplanned hospital admissions during follow-up

**Fig. 2:**
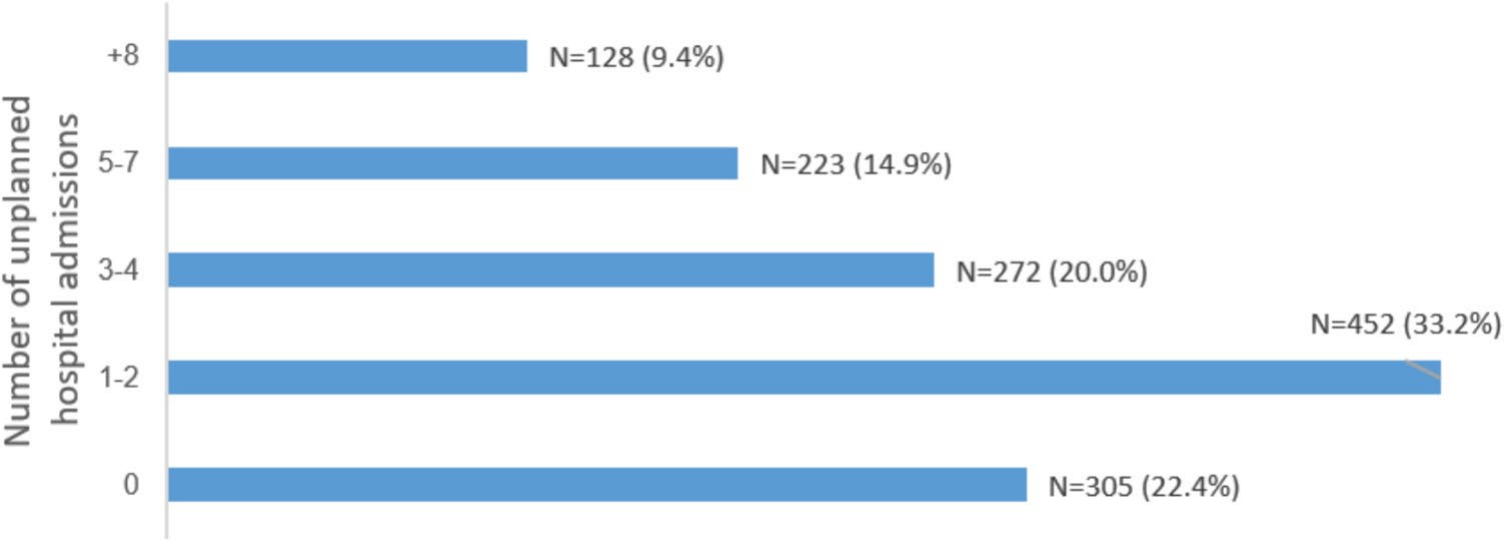
The distribution of the study participants by the total number of unplanned hospital admissions during follow-up

The mean (standard deviation) age of the patients was 70.7 (11.3) years; 72.5% of them were men, 64.3% had reduced left ventricular ejection fraction (LVEF < 40%), and 38.3% had hospital admissions for decompensated HF within the previous 2 months. During 3421 patient-years, there were 5192 eligible hospital admissions, of which 4251 (81.9%) were unplanned. Discharge summaries of all hospital admissions were available for analysis. Figure 1 presents the ten most frequent causes of unplanned hospitalizations and Fig. 2 shows the distribution of the study participants by the total number of unplanned hospital admissions they experienced during follow-up. HF was the main cause for 33.3% of admissions, followed by coronary artery disease (8.4%) and arrhythmia or conduction disturbance (7%). Three hundred and five (22.4%) patients did not experience an unplanned hospital admission during follow-up and 450 patients died (33.1%).

We identified five patient clusters with distinctive unplanned hospital admission profiles. Table 1 describes the five patient clusters according to the main most frequent causes of unplanned hospital admissions they experienced during follow-up, and the mean number of hospitalizations from each cause. The information on the mean number of admissions for the complete list of diagnosis categories (*N* = 43) is presented in Table 2 of Supplementary Information 1.

**Table 2:** Distribution of unplanned hospital admissions and in-hospital days by patient subgroups

	Patients, *N* (%)	Unplanned admissions, *N* (%)	Unplanned in-hospital days, *N* (%)
Patients with no unplanned admissions	305 (22.4)	0	0
Cluster 1	307 (22.6)	1066 (25.1)	6778 (26.2)
Cluster 2	528 (38.8)	1413 (33.2)	8242 (31.9)
Cluster 3	49 (3.6)	340 (8.0)	2021 (7.8)
Cluster 4	115 (8.5)	753 (17.7)	5019 (19.4)
Cluster 5	56 (4.1)	679 (16.0)	3805 (14.7)
Total, *N* (%)	1360 (100)	4251 (100)	25,865 (100)

The first cluster included 307 (22.6%) patients, with a mean number of 3.5 unplanned admissions per patient during follow-up. HF was the most frequent admission cause in this subgroup, followed by acute bronchitis/pneumonia. The second cluster included the highest number of patients (*N* = 528; 38.8%), with a mean number of 2.7 unplanned admissions per patient during follow-up. There was no single prominent cause of admission among patients included in this cluster. The third cluster included 49 (3.6%) patients, with a mean number of 6.9 unplanned admissions per patient during follow-up. Coronary artery disease and arrhythmia/conduction disturbance were the most frequent main causes of admission among these patients, followed by HF. The fourth and fifth clusters included 115 (8.5%) and 56 (4.1%) patients, respectively, with a mean number of 6.6 and 12.1 unplanned admissions per patient during follow-up. HF was the most frequent main admission cause in both clusters, followed by anemia in the fourth cluster, and by coronary artery disease, arrhythmia/conduction disturbance, acute bronchitis/pneumonia, and chronic lung disease in the fifth cluster.

While the third, fourth, and fifth clusters accounted, together, for only 16% of the total patient sample, their share in the total number of unplanned hospital admissions and in-hospital days was 42% (Table 2).

Table 3 shows the baseline and follow-up characteristics of the entire cohort and univariate comparisons between the patient subgroups, i.e., patients who did not experience an unplanned hospital admission during follow-up, and the five clusters with distinctive unplanned hospital admission profiles.

**Table 3:** Univariate comparisons of baseline and follow-up characteristics by patient unplanned hospital admission profile

Baseline characteristics	All patients *N* = 1360	Patient subgroups						p*
		No admission *N* = 305	Cluster 1 *N* = 307	Cluster 2 *N* = 528	Cluster 3 *N* = 49	Cluster 4 *N* = 115	Cluster 5 *N* = 56	
Age, years, mean (SD)	70.8 (11.3)	68.1 (12.1)	72.3 (10.9)	71.5 (11.2)	69.3 (10.4)	72.1 (10.6)	68.1 (11.1)	< 0.0001
Female, *N* (%)	374 (27.5)	84 (27.5)	81 (26.4)	151 (28.6)	12 (24.5)	34 (29.6)	12 (21.4)	0.85
Study arm: disease management, *N* (%)	682 (50.1)	154 (50.5)	147 (47.9)	274 (51.9)	23 (46.9)	59 (51.3)	25 (44.6)	0.82
LVEF ≥ 40%, *N* (%)	480 (35.7)	94 (31.1)	136 (44.9)	174 (33.4)	14 (29.2)	42 (37.2)	20 (35.7)	0.0066
6-min. walking distance, meters, median (IQR)	180 (81, 291)	240 (108, 333)	150 (72, 275.5)	182 (80, 290)	166 (100, 282)	162 (70, 270)	180 (66, 265)	< 0.0001
Hemoglobin, gr/dL, mean (SD)	12.6 (1.9)	13.1 (1.6)	12.6 (1.7)	12.7 (1.7)	12.6 (1.4)	12.1 (1.7)	12.2 (1.7)	< 0.0001
eGFR < 60 ml/min/1.73 m^2^, *N* (%)	755 (55.5)	127 (42.5)	192 (64.0)	291 (56.3)	22 (44.9)	87 (76.3)	36 (66.7)	< 0.0001
Charlson’s comorbidity score, median (IQR)	4 (3, 6)	3 (2, 5)	5 (4, 6)	4 (3, 5)	4 (3, 6)	5 (4, 6)	4 (4, 6)	< 0.0001
Treatment with loop diuretic at baseline, vs. no treatment	1208 (88.8)	248 (81.3)	286 (93.2)	461 (87.3)	45 (91.8)	113 (98.3)	55 (98.2)	< 0.0001
Main cause of HF: CAD, *N* (%)	966 (71.0)	192 (62.9)	221 (72.0)	378 (71.6)	42 (85.7)	90 (78.3)	42 (76.8)	0.0020
Main cause of HF: valvular disease, *N* (%)	313 (23.0)	53 (17.4)	86 (28.0)	124 (23.5)	8 (16.3)	29 (25.2)	13 (23.1)	0.043
Recent (≤ 2 mo.) hospital admission for HF exacerbation, *N* (%)	521 (38.3)	102 (33.4)	149 (48.5)	177 (33.5)	23 (46.9)	42 (36.5)	28 (50.0)	< 0.0001
Atrial fibrillation, *N* (%)	512 (37.6)	102 (33.4)	128 (41.7)	194 (36.7)	19 (38.8)	49 (42.6)	20 (35.7)	0.31
ICD at baseline, *N* (%)	215 (15.8)	45 (14.7)	43 (14.0)	80 (15.1)	17 (34.7)	22 (19.1)	8 (14.3)	0.0089
Length of follow-up, days, median (IQR)	980 (663, 1341)	961 (726, 1260)	933 (528, 1276)	988 (705, 1359)	1192 (824, 1506)	995 (687, 1401)	1012 (637, 1292)	0.0081
Number of unplanned hospital admissions during follow-up, median (IQR)	2 (1, 4)	–	3 (1, 5)	2 (1, 3)	6 (5, 9)	6 (4, 8)	11 (8, 16)	< 0.0001
Died during follow-up, *N* (%)	450 (33.0)	26 (8.5)	144 (46.9)	168 (31.8)	18 (36.7)	63 (54.8)	31 (55.4)	< 0.0001

*LVEF* Left ventricular ejection fraction; *eGFR* estimated glomerular filtration rate, ml/min/1.73 m^2^; *CAD* coronary artery disease; *HF*: heart failure; *ICD* implantable cardioverter defibrillator; *IQR* interquartile range (25th, 75th percentiles); *SD* standard deviation

*Tested with the Chi-square statistic for contingency tables and the Kruskal‒Wallis test for continuous variables

Patients in clusters 1, 2, and 4 were approximately 3–4 years older than patients in clusters 3 and 5 and patients who had not experienced an unplanned admission during follow-up. Patients in all clusters had higher baseline Charlson comorbidity scores, lower hemoglobin levels, and shorter 6-min walking distances compared to patients who did not experience an unplanned admission during follow-up.

More than half of the patients in the fourth and fifth clusters died during follow-up, while patients who did not experience an unplanned admission during follow-up had the best prognosis, with a mortality rate of 8.5% (Table 3).

The median length of follow-up among patients in the first patient cluster was 0.6 years shorter than that for patients in the third cluster; otherwise, the length of follow-up did not differ significantly among patient subgroups. The proportion of patients assigned to the disease management intervention and the proportion of women was similar across patient subgroups. Likewise, the proportion of patients with an implanted pacemaker or treated with beta-adrenergic blockers, mineralocorticoid receptor antagonists, and angiotensin-converting enzyme inhibitors or angiotensin receptor blockers at baseline did not differ significantly by patient subgroup in univariate analysis (Table 1 in Supplementary Information 2).

Table 4 shows the baseline characteristics associated with the five patient clusters in multinomial logistic regression analysis, where patients who did not experience an unplanned admission were the reference category.

**Table 4:** Baseline predictors for unplanned hospital admission profiles during follow-up: multinomial logistic regression model

Baseline characteristics	Odds ratio (95% confidence interval)					p*
	Cluster 1	Cluster 2	Cluster 3	Cluster 4	Cluster 5	
Age (10-yr increment)	1.05 (0.89, 1.25)	1.11 (0.96, 1.28)	0.94 (0.69, 1.28)	1.01 (0.80, 1.28)	**0.71 (0.53, 0.95)**	0.053
Female vs. male	0.78 (0.50, 1.21)	0.99 (0.68, 1.45)	0.90 (0.40, 2.04)	0.91 (0.51, 1.60)	0.58 (0.26, 1.28)	0.65
6-min. walk-test (50 m increment)	**0.89 (0.82, 0.95)**	**0.92 (0.87, 0.98)**	**0.86 (0.76, 0.98)**	**0.87 (0.79, 0.96)**	**0.86 (0.76, 0.97)**	**0.0066**
Baseline hemoglobin, gr/dL (1 unit increment)	0.96 (0.86, 1.07)	0.91 (0.83, 1.01)	0.83 (0.68, 1.03)	**0.75 (0.64, 0.87)**	**0.75 (0.61, 0.92)**	**0.0009**
Charlson’s comorbidity score (1-point increment)	**1.26 (1.12, 1.41)**	**1.13 (1.02, 1.25)**	0.99 (0.80, 1.24)	**1.28 (1.10, 1.49)**	1.18 (0.96, 1.44)	**0.0010**
Treatment with loop diuretic at baseline, vs. no treatment	**2.14 (1.23, 3.75)**	1.37 (0.90, 2.07)	2.23 (0.74, 6.67)	**19.61 (2.65, 145.35)**	**9.79 (1.30, 73.53)**	**0.0022**
Main cause of HF: CAD vs. other	1.18 (0.78, 1.79)	1.27 (0.89, 1.83)	**3.62 (1.44, 9.11)**	1.69 (0.94, 3.06)	1.53 (0.71, 3.28)	**0.084**
Recent (≤ 2 mo.) hospital admission for HF exacerbation	**0.67 (0.47, 0.95)**	1.15 (0.84, 1.58)	0.56 (0.29, 1.06)	1.22 (0.76, 1.98)	0.64 (0.35, 1.18)	**0.0019**
LVEF = > 40% vs. < 40%	**1.88 (1.28, 2.77)**	1.14 (0.81, 1.62)	1.34 (0.64, 2.83)	1.41 (0.84, 2.38)	1.34 (0.68, 2.62)	**0.027**
ICD at baseline	1.31 (0.79, 2.20)	1.27 (0.82, 1.97)	**3.79 (1.79, 8.02)**	**2.12 (1.13, 3.97)**	0.99 (0.40, 2.46)	**0.0079**

The bold font in point at associations found statistically significant in the multivariable models

The model was adjusted for the assigned treatment arm, which was not found to be statistically significant (*p* = 0.53), and the follow-up period. The reference group in the model included patients who did not have an unplanned admission during follow-up

*HF* Heart failure; *CAD* coronary artery disease; *LVEF* left ventricular ejection fraction; *ICD* implantable cardioverter defibrillator

*Type-3 comparisons

Compared to patients who did not experience an unplanned admission during follow-up, patients in all five clusters were more likely to have shorter 6-min walking distances. In addition, patients in clusters 1, 2, 4, and 5 were more likely to have a higher Charlson’s comorbidity score, and patients in clusters 4 and 5 had a very high likelihood of receiving loop diuretic treatment before the start of follow-up.

Patients in cluster 1 were more likely to have mildly reduced or preserved LVEF (≥ 40%) and were less likely to have a recent hospital admission for HF exacerbation before recruitment.

Patients in cluster 3 were 3.6 times as likely to have HF due to ischemic heart disease as patients who did not experience an unplanned admission during follow-up.

Patients in clusters 3 and 4 were more likely to have an implantable cardioverter defibrillator at baseline than patients who did not experience an unplanned admission during follow-up.

Lower baseline hemoglobin levels were significantly associated with clusters 4 and 5.

Finally, patients in cluster 5 were more likely to be younger than patients who did not have an unplanned admission during follow-up.

Altogether, the optimal classification erroneously assigned seven (3.2%) patients in subgroups 3–5 (i.e. patients with high burden or hospital admissions) to the reference group who did not experience an unplanned hospital admission during follow-up. On the other hand, 36 (11.8%) patients who did not experience an unplanned hospital admission during follow-up were erroneously assigned to subgroups 3, 4, or 5. Complete information on the model discrimination appears in Supplementary Information 3.

## Discussion

In this study, we identified five unique patient subgroups (clusters) with different profiles of unplanned hospital admissions during follow-up. Almost half of these admissions occurred in a small fraction of patients who are natural candidates for targeted interventions, aiming to reduce the burden of recurrent hospitalizations. In addition, we found that approximately one-fifth of the patients did not experience an unplanned hospitalization during follow-up. These patients, characterized by lower baseline comorbidity, higher hemoglobin level, better functional capacity, and lower likelihood for loop diuretic treatment, are not candidates for intensive interventions. We found that almost 40% of the patients with HF had, on average, approximately one unplanned admission per year for various reasons, without any prominent admission cause. These patients, who had slightly higher baseline comorbidity score and lower functional capacity than patients who did not experience hospitalization during follow-up, can be managed jointly by a primary physician and consultant cardiologist. Finally, approximately one-fifth of patients, having slightly more than one admission per year, were hospitalized mainly for HF. These patients differed from the other patient subgroups by a higher likelihood of having mildly reduced or preserved LVEF. In addition to joint management by a primary physician and consultant cardiologist, these patients may benefit from treatment with sodium-glucose cotransporter-2 (SGLT-2) inhibitors, recently recommended for patients with mildly reduced or preserved LVEF [19, 20].

Previous studies used latent class analysis and cluster analysis to identify unique clinical and demographic characteristics associated with health outcomes among patients with HF. Guela et al. [21] identified five comorbidity clusters that predicted health outcomes among HF patients using a large administrative data warehouse. They found that patients in the diabetes, obesity, and vascular disease comorbidity group had the highest hazard ratio for first hospital admission and death during follow-up compared to patients with a low-comorbidity profile. Recently, Murray et al. [22] identified four distinct patient clusters associated with health outcomes among HF patients with preserved LVEF using data from the ASCENT-HF trial. They found that patients who were predominantly White or Asian, with high rates of atrial fibrillation, high blood natriuretic peptide level, low systolic blood pressure, and high baseline heart rate, had the highest risk of death or first hospital admission within 30 days. However, both studies did not look at predictors associated with the burden of recurrent hospital admissions by specific causes, which is a prerequisite for assigning patients to effective interventions.

The current study confirms previous observations, showing that chronic HF is associated with poor prognosis [1, 2] and a heavy burden of recurrent unplanned hospital admissions [4–7], most of which are not due to HF exacerbation [6–10].

Our study has a few limitations. The study sample included HF patients recruited in a clinical trial, with pre-specified inclusion and exclusion criteria. Patients with mild HF (New York Heart Association functional class I) and bedridden patients were excluded. However, patients with mild HF have a low burden of unplanned hospital admissions, while most bedridden HF patients are included in home-care programs. Patients included in our study were recruited before the introduction of angiotensin receptor/neprilysin inhibitors (ARNi) and SGLT-2 inhibitors, recommended in contemporary guidelines for the treatment of patients with chronic HF [19, 20]. A meta-analysis of the DAPA-HF and DELIVER trial data showed that SGLT-2 inhibitor treatment was associated with a lower hospital admission rate for HF [23]. ARNi was more effective than angiotensin-converting enzyme inhibitor treatment in reducing recurrent hospital admissions for HF in the PARADIGM-HF trial [24]. Nevertheless, most recurrent hospital admissions among HF patients are not due to HF exacerbations [6–10], and there are yet no published data on the effect of both treatments on hospital admissions for causes other than HF. The change in medical care recommended for HF patients may increase the proportion of hospital admissions for causes other than HF even further.

Our study has several strengths, including a systematic collection of pertinent information on patient characteristics (e.g., NYHA classification, LVEF, and 6-min walk test) and hospital admission causes. This information is not always available in research that relies on administrative patient databases.

## Conclusions

The current study offers a method to identify subgroups of HF patients with distinctive unplanned hospital admission profiles (by causes and frequency) and the baseline clinical predictors associated with subgroup membership. This method can be applied and fine-tuned further to contemporary patient cohorts. It can further guide the design of personalized interventions to reduce the burden of unplanned hospital admissions and direct these interventions to patients most likely to benefit from them. The efficacy of such personalized interventions should be tested in randomized trials.

## Supplementary Information

Below is the link to the electronic supplementary material.

Supplementary file1 (DOCX 47 KB)

Supplementary file2 (DOCX 15 KB)

Supplementary file3 (DOCX 28 KB)

## Data Availability

Not applicable.

## Abbreviations

HF: Heart failure
CAD: Coronary artery disease
ICD: Implantable cardioverter defibrillator
LVEF: Left ventricular ejection fraction
SGLT-2i: Sodium-glucose cotransporter-2 inhibitors
ARNi: Angiotensin receptor-neprilysin inhibitor
eGFR: Estimated glomerular filtration rate
SD: Standard deviation
IQR: Interquartile range
NYHA: New York heart association
