# Supplementary material for: Causes and predictors of recurrent unplanned hospital admissions in heart failure patients: a cohort study
Source: Intern Emerg Med. 2024 Aug 18;19(8):2213–21. doi: 10.1007/s11739-024-03740-2 (PMC11582252; doi:10.1007/s11739-024-03740-2)
Supplement: Supplementary file 1 — Supplementary file1 (DOCX 47 KB) [file 11739_2024_3740_MOESM1_ESM.docx]

Supplementary Information 1

**Causes and Predictors of Recurrent Unplanned Hospital Admissions in Heart Failure Patients: A Cohort Study**

Ofra Kalter-Leibovici^1, 2*^, Havi Murad^1^, Arnona Ziv^1^, Tomer Keidan^3^, Alon Orion^4^, Yoav Afel^5^, Harel Gilutz^6^, Dov Freimark^5^, Rachel Klibansky-Marom^1^, Laurence Freedman^1^, Haim Silber^7^.

SI Table 1. Groups of main causes for unplanned hospital admissions

| Group number | Description |
| --- | --- |
| 1 | Cardiovascular risk factors and their complications |
| 2 | Coronary artery disease |
| 3 | Cardiac arrhythmia/conduction disturbances |
| 4 | Heart failure |
| 5 | Heart valve disease |
| 6 | Other heart disease |
| 7 | Cerebrovascular disease (stroke; transient cerebral ischemia) |
| 8 | Peripheral vascular disease |
| 9 | Thromboembolic events |
| 10 | Other vascular diseases |
| 11 | Venous and lymphatic system disorders |
| 12 | Lower limb ulcer |
| 13 | Renal failure/renal disease |
| 14 | Electrolyte and fluid imbalance |
| 15 | Urological diseases |
| 16 | Obstructive/restrictive lung disease |
| 17 | Respiratory failure |
| 18 | Other acute respiratory symptoms |
| 19 | Upper respiratory tract infections |
| 20 | Bronchitis and pneumonia |
| 21 | Other acute infections (e.g. urinary tract infection, acute gastroenteritis) |
| 22 | Cellulitis and erysipelas |
| 23 | Bacteremia and sepsis |
| 24 | Accidents and other trauma |
| 25 | Falls, contusion, fractures, including treatment complications |
| 26 | Vertigo |
| 27 | Hypotension |
| 28 | Syncope |
| 29 | Other neurological disorders |
| 30 | Anemia |
| 31 | Major bleeding events (gastrointestinal, intra-cranial and other) |
| 32 | Platelet or other hematological disorders |
| 33 | Malignancy and myeloproliferative disorders |
| 34 | Ear, nose and throat disorders |
| 35 | Endocrine disorders |
| 36 | Gastrointestinal disorders |
| 37 | Ophthalmological disorders |
| 38 | Dermatological disorders, including tumors and inflammation |
| 39 | Other musculoskeletal disorders |
| 40 | Cognitive and mental disorders |
| 41 | General deterioration, weight loss |
| 42 | Side effects of drugs |
| 43 | Other disorders, complications |

SI Table 2. The distribution of the mean number of unplanned hospital admissions by main admission diagnosis group and patient cluster.

| **Cluster** | **N Obs** | **Main admission cause*** | **N** | **Mean** | **Std Dev** | **Lower Quartile** | **Median** | **Upper Quartile** | **Minimum** | **Maximum** |
| --- | --- | --- | --- | --- | --- | --- | --- | --- | --- | --- |
| 1 | 307 | 1 2 3 4 5 6 7 8 9 10 11 12 13 14 15 16 17 18 19 20 21 22 23 24 25 26 27 28 29 30 31 32 33 34 35 36 37 38 39 40 41 42 43 | 307 307 307 307 307 307 307 307 307 307 307 307 307 307 307 307 307 307 307 307 307 307 307 307 307 307 307 307 307 307 307 307 307 307 307 307 307 307 307 307 307 307 307 | 0.0 0.2 0.1 1.3 0.0 0.0 0.1 0.0 0.1 0.0 0.0 0.0 0.1 0.0 0.0 0.4 0.1 0.1 0.1 0.0 0.1 0.0 0.0 0.0 0.1 0.0 0.0 0.0 0.0 0.0 0.0 0.0 0.0 0.0 0.0 0.1 0.0 0.0 0.0 0.0 0.0 0.1 0.0 | 0.2 0.5 0.3 0.7 0.3 0.1 0.3 0.2 0.3 0.1 0.1 0.2 0.3 0.1 0.2 0.8 0.4 0.3 0.3 0.2 0.3 0.1 0.1 0.0 0.6 0.1 0.2 0.1 0.2 0.2 0.1 0.2 0.1 0.1 0.1 0.3 0.2 0.1 0.2 0.1 0.2 0.3 0.0 | 0.0 0.0 0.0 1.0 0.0 0.0 0.0 0.0 0.0 0.0 0.0 0.0 0.0 0.0 0.0 0.0 0.0 0.0 0.0 0.0 0.0 0.0 0.0 0.0 0.0 0.0 0.0 0.0 0.0 0.0 0.0 0.0 0.0 0.0 0.0 0.0 0.0 0.0 0.0 0.0 0.0 0.0 0.0 | 0.0 0.0 0.0 1.0 0.0 0.0 0.0 0.0 0.0 0.0 0.0 0.0 0.0 0.0 0.0 0.0 0.0 0.0 0.0 0.0 0.0 0.0 0.0 0.0 0.0 0.0 0.0 0.0 0.0 0.0 0.0 0.0 0.0 0.0 0.0 0.0 0.0 0.0 0.0 0.0 0.0 0.0 0.0 | 0.0 0.0 0.0 2.0 0.0 0.0 0.0 0.0 0.0 0.0 0.0 0.0 0.0 0.0 0.0 1.0 0.0 0.0 0.0 0.0 0.0 0.0 0.0 0.0 0.0 0.0 0.0 0.0 0.0 0.0 0.0 0.0 0.0 0.0 0.0 0.0 0.0 0.0 0.0 0.0 0.0 0.0 0.0 | 0.0 0.0 0.0 0.0 0.0 0.0 0.0 0.0 0.0 0.0 0.0 0.0 0.0 0.0 0.0 0.0 0.0 0.0 0.0 0.0 0.0 0.0 0.0 0.0 0.0 0.0 0.0 0.0 0.0 0.0 0.0 0.0 0.0 0.0 0.0 0.0 0.0 0.0 0.0 0.0 0.0 0.0 0.0 | 2.0 3.0 2.0 4.0 3.0 1.0 3.0 2.0 2.0 1.0 1.0 2.0 2.0 1.0 1.0 5.0 2.0 2.0 2.0 2.0 2.0 1.0 2.0 0.0 5.0 1.0 1.0 1.0 1.0 1.0 1.0 2.0 1.0 1.0 1.0 3.0 2.0 1.0 2.0 1.0 2.0 2.0 0.0 |
| 2 | 528 | 1 2 3 4 5 6 7 8 9 10 11 12 13 14 15 16 17 18 19 20 21 22 23 24 25 26 27 28 29 30 31 32 33 34 35 36 37 38 39 40 41 42 43 | 528 528 528 528 528 528 528 528 528 528 528 528 528 528 528 528 528 528 528 528 528 528 528 528 528 528 528 528 528 528 528 528 528 528 528 528 528 528 528 528 528 528 528 | 0.0 0.2 0.2 0.2 0.0 0.0 0.1 0.0 0.1 0.0 0.0 0.0 0.1 0.0 0.1 0.1 0.1 0.1 0.1 0.1 0.1 0.0 0.0 0.0 0.0 0.0 0.1 0.0 0.0 0.0 0.0 0.0 0.0 0.0 0.0 0.2 0.1 0.0 0.0 0.0 0.0 0.0 0.0 | 0.3 0.5 0.5 0.5 0.1 0.2 0.3 0.2 0.7 0.1 0.2 0.2 0.3 0.1 0.3 0.3 0.4 0.3 0.3 0.3 0.4 0.2 0.1 0.1 0.2 0.2 0.5 0.1 0.2 0.3 0.1 0.0 0.1 0.1 0.0 0.5 0.3 0.3 0.2 0.1 0.2 0.2 0.1 | 0.0 0.0 0.0 0.0 0.0 0.0 0.0 0.0 0.0 0.0 0.0 0.0 0.0 0.0 0.0 0.0 0.0 0.0 0.0 0.0 0.0 0.0 0.0 0.0 0.0 0.0 0.0 0.0 0.0 0.0 0.0 0.0 0.0 0.0 0.0 0.0 0.0 0.0 0.0 0.0 0.0 0.0 0.0 | 0.0 0.0 0.0 0.0 0.0 0.0 0.0 0.0 0.0 0.0 0.0 0.0 0.0 0.0 0.0 0.0 0.0 0.0 0.0 0.0 0.0 0.0 0.0 0.0 0.0 0.0 0.0 0.0 0.0 0.0 0.0 0.0 0.0 0.0 0.0 0.0 0.0 0.0 0.0 0.0 0.0 0.0 0.0 | 0.0 0.0 0.0 0.0 0.0 0.0 0.0 0.0 0.0 0.0 0.0 0.0 0.0 0.0 0.0 0.0 0.0 0.0 0.0 0.0 0.0 0.0 0.0 0.0 0.0 0.0 0.0 0.0 0.0 0.0 0.0 0.0 0.0 0.0 0.0 0.0 0.0 0.0 0.0 0.0 0.0 0.0 0.0 | 0.0 0.0 0.0 0.0 0.0 0.0 0.0 0.0 0.0 0.0 0.0 0.0 0.0 0.0 0.0 0.0 0.0 0.0 0.0 0.0 0.0 0.0 0.0 0.0 0.0 0.0 0.0 0.0 0.0 0.0 0.0 0.0 0.0 0.0 0.0 0.0 0.0 0.0 0.0 0.0 0.0 0.0 0.0 | 3.0 2.0 2.0 2.0 1.0 3.0 2.0 3.0 10.0 1.0 2.0 1.0 2.0 2.0 2.0 2.0 3.0 2.0 2.0 3.0 2.0 2.0 1.0 2.0 1.0 3.0 4.0 1.0 2.0 3.0 2.0 1.0 1.0 2.0 1.0 4.0 2.0 6.0 2.0 1.0 2.0 1.0 2.0 |
| 3 | 49 | 1 2 3 4 5 6 7 8 9 10 11 12 13 14 15 16 17 18 19 20 21 22 23 24 25 26 27 28 29 30 31 32 33 34 35 36 37 38 39 40 41 42 43 | 49 49 49 49 49 49 49 49 49 49 49 49 49 49 49 49 49 49 49 49 49 49 49 49 49 49 49 49 49 49 49 49 49 49 49 49 49 49 49 49 49 49 49 | 0.1 2.0 2.1 0.9 0.0 0.1 0.1 0.1 0.0 0.0 0.0 0.0 0.2 0.0 0.0 0.2 0.1 0.1 0.0 0.0 0.0 0.0 0.0 0.0 0.0 0.1 0.1 0.0 0.0 0.0 0.0 0.0 0.0 0.0 0.0 0.1 0.0 0.0 0.1 0.1 0.0 0.1 0.0 | 0.3 1.9 2.1 1.1 0.1 0.5 0.3 0.2 0.2 0.1 0.0 0.1 0.4 0.2 0.2 0.4 0.2 0.5 0.2 0.2 0.2 0.2 0.0 0.0 0.1 0.3 0.2 0.1 0.0 0.1 0.1 0.0 0.0 0.1 0.0 0.3 0.1 0.3 0.5 0.2 0.2 0.3 0.0 | 0.0 0.0 0.0 0.0 0.0 0.0 0.0 0.0 0.0 0.0 0.0 0.0 0.0 0.0 0.0 0.0 0.0 0.0 0.0 0.0 0.0 0.0 0.0 0.0 0.0 0.0 0.0 0.0 0.0 0.0 0.0 0.0 0.0 0.0 0.0 0.0 0.0 0.0 0.0 0.0 0.0 0.0 0.0 | 0.0 2.0 2.0 0.0 0.0 0.0 0.0 0.0 0.0 0.0 0.0 0.0 0.0 0.0 0.0 0.0 0.0 0.0 0.0 0.0 0.0 0.0 0.0 0.0 0.0 0.0 0.0 0.0 0.0 0.0 0.0 0.0 0.0 0.0 0.0 0.0 0.0 0.0 0.0 0.0 0.0 0.0 0.0 | 0.0 3.0 3.0 1.0 0.0 0.0 0.0 0.0 0.0 0.0 0.0 0.0 0.0 0.0 0.0 0.0 0.0 0.0 0.0 0.0 0.0 0.0 0.0 0.0 0.0 0.0 0.0 0.0 0.0 0.0 0.0 0.0 0.0 0.0 0.0 0.0 0.0 0.0 0.0 0.0 0.0 0.0 0.0 | 0.0 0.0 0.0 0.0 0.0 0.0 0.0 0.0 0.0 0.0 0.0 0.0 0.0 0.0 0.0 0.0 0.0 0.0 0.0 0.0 0.0 0.0 0.0 0.0 0.0 0.0 0.0 0.0 0.0 0.0 0.0 0.0 0.0 0.0 0.0 0.0 0.0 0.0 0.0 0.0 0.0 0.0 0.0 | 2.0 10.0 10.0 4.0 1.0 3.0 1.0 1.0 1.0 1.0 0.0 1.0 2.0 1.0 1.0 1.0 1.0 3.0 1.0 1.0 1.0 1.0 0.0 0.0 1.0 1.0 1.0 1.0 0.0 1.0 1.0 0.0 0.0 1.0 0.0 1.0 1.0 2.0 3.0 1.0 1.0 1.0 0.0 |
| 4 | 115 | 1 2 3 4 5 6 7 8 9 10 11 12 13 14 15 16 17 18 19 20 21 22 23 24 25 26 27 28 29 30 31 32 33 34 35 36 37 38 39 40 41 42 43 | 115 115 115 115 115 115 115 115 115 115 115 115 115 115 115 115 115 115 115 115 115 115 115 115 115 115 115 115 115 115 115 115 115 115 115 115 115 115 115 115 115 115 115 | 0.1 0.2 0.2 3.6 0.0 0.0 0.1 0.1 0.0 0.0 0.0 0.0 0.0 0.0 0.1 0.2 0.2 0.1 0.1 0.1 0.2 0.0 0.0 0.0 0.1 0.0 0.3 0.0 0.1 0.0 0.0 0.0 0.0 0.0 0.0 0.2 0.1 0.0 0.0 0.0 0.0 0.0 0.0 | 0.2 0.5 0.5 1.0 0.2 0.2 0.4 0.3 0.2 0.2 0.0 0.0 0.2 0.2 0.2 0.5 0.8 0.3 0.5 0.3 0.5 0.2 0.0 0.0 0.2 0.2 1.3 0.0 0.5 0.3 0.1 0.0 0.1 0.0 0.0 0.6 0.3 0.1 0.2 0.1 0.2 0.2 0.1 | 0.0 0.0 0.0 3.0 0.0 0.0 0.0 0.0 0.0 0.0 0.0 0.0 0.0 0.0 0.0 0.0 0.0 0.0 0.0 0.0 0.0 0.0 0.0 0.0 0.0 0.0 0.0 0.0 0.0 0.0 0.0 0.0 0.0 0.0 0.0 0.0 0.0 0.0 0.0 0.0 0.0 0.0 0.0 | 0.0 0.0 0.0 3.0 0.0 0.0 0.0 0.0 0.0 0.0 0.0 0.0 0.0 0.0 0.0 0.0 0.0 0.0 0.0 0.0 0.0 0.0 0.0 0.0 0.0 0.0 0.0 0.0 0.0 0.0 0.0 0.0 0.0 0.0 0.0 0.0 0.0 0.0 0.0 0.0 0.0 0.0 0.0 | 0.0 0.0 0.0 4.0 0.0 0.0 0.0 0.0 0.0 0.0 0.0 0.0 0.0 0.0 0.0 0.0 0.0 0.0 0.0 0.0 0.0 0.0 0.0 0.0 0.0 0.0 0.0 0.0 0.0 0.0 0.0 0.0 0.0 0.0 0.0 0.0 0.0 0.0 0.0 0.0 0.0 0.0 0.0 | 0.0 0.0 0.0 0.0 0.0 0.0 0.0 0.0 0.0 0.0 0.0 0.0 0.0 0.0 0.0 0.0 0.0 0.0 0.0 0.0 0.0 0.0 0.0 0.0 0.0 0.0 0.0 0.0 0.0 0.0 0.0 0.0 0.0 0.0 0.0 0.0 0.0 0.0 0.0 0.0 0.0 0.0 0.0 | 1.0 3.0 2.0 7.0 1.0 1.0 2.0 2.0 1.0 1.0 0.0 0.0 1.0 1.0 1.0 2.0 5.0 2.0 3.0 2.0 3.0 2.0 0.0 0.0 1.0 2.0 10.0 0.0 3.0 2.0 1.0 0.0 1.0 0.0 0.0 3.0 2.0 1.0 1.0 1.0 1.0 2.0 1.0 |
| 5 | 56 | 1 2 3 4 5 6 7 8 9 10 11 12 13 14 15 16 17 18 19 20 21 22 23 24 25 26 27 28 29 30 31 32 33 34 35 36 37 38 39 40 41 42 43 | 56 56 56 56 56 56 56 56 56 56 56 56 56 56 56 56 56 56 56 56 56 56 56 56 56 56 56 56 56 56 56 56 56 56 56 56 56 56 56 56 56 56 56 | 0.0 0.7 0.4 7.8 0.0 0.2 0.2 0.1 0.4 0.1 0.0 0.1 0.1 0.1 0.1 0.4 0.1 0.2 0.1 0.1 0.1 0.0 0.0 0.0 0.0 0.1 0.1 0.0 0.0 0.0 0.0 0.0 0.0 0.0 0.0 0.2 0.0 0.1 0.1 0.1 0.0 0.1 0.0 | 0.3 1.2 0.9 2.3 0.0 0.9 0.5 0.2 0.9 0.6 0.2 0.2 0.3 0.2 0.3 1.0 0.6 0.6 0.2 0.3 0.2 0.1 0.2 0.0 0.1 0.3 0.4 0.0 0.0 0.1 0.2 0.0 0.0 0.0 0.0 0.5 0.2 0.3 0.4 0.3 0.0 0.3 0.1 | 0.0 0.0 0.0 6.0 0.0 0.0 0.0 0.0 0.0 0.0 0.0 0.0 0.0 0.0 0.0 0.0 0.0 0.0 0.0 0.0 0.0 0.0 0.0 0.0 0.0 0.0 0.0 0.0 0.0 0.0 0.0 0.0 0.0 0.0 0.0 0.0 0.0 0.0 0.0 0.0 0.0 0.0 0.0 | 0.0 0.0 0.0 7.0 0.0 0.0 0.0 0.0 0.0 0.0 0.0 0.0 0.0 0.0 0.0 0.0 0.0 0.0 0.0 0.0 0.0 0.0 0.0 0.0 0.0 0.0 0.0 0.0 0.0 0.0 0.0 0.0 0.0 0.0 0.0 0.0 0.0 0.0 0.0 0.0 0.0 0.0 0.0 | 0.0 1.0 0.5 9.0 0.0 0.0 0.0 0.0 0.0 0.0 0.0 0.0 0.0 0.0 0.0 1.0 0.0 0.0 0.0 0.0 0.0 0.0 0.0 0.0 0.0 0.0 0.0 0.0 0.0 0.0 0.0 0.0 0.0 0.0 0.0 0.0 0.0 0.0 0.0 0.0 0.0 0.0 0.0 | 0.0 0.0 0.0 5.0 0.0 0.0 0.0 0.0 0.0 0.0 0.0 0.0 0.0 0.0 0.0 0.0 0.0 0.0 0.0 0.0 0.0 0.0 0.0 0.0 0.0 0.0 0.0 0.0 0.0 0.0 0.0 0.0 0.0 0.0 0.0 0.0 0.0 0.0 0.0 0.0 0.0 0.0 0.0 | 2.0 5.0 4.0 14.0 0.0 6.0 3.0 1.0 3.0 4.0 1.0 1.0 1.0 1.0 1.0 6.0 4.0 2.0 1.0 1.0 1.0 1.0 1.0 0.0 1.0 2.0 2.0 0.0 0.0 1.0 1.0 0.0 0.0 0.0 0.0 3.0 1.0 2.0 2.0 2.0 0.0 2.0 1.0 |

*according to diagnosis groups described in SI Table-1.
