# Supplementary material for: Causes and predictors of recurrent unplanned hospital admissions in heart failure patients: a cohort study
Source: Intern Emerg Med. 2024 Aug 18;19(8):2213–21. doi: 10.1007/s11739-024-03740-2 (PMC11582252; doi:10.1007/s11739-024-03740-2)
Supplement: Supplementary file 2 — Supplementary file2 (DOCX 15 KB) [file 11739_2024_3740_MOESM2_ESM.docx]

**Causes and Predictors of Recurrent Unplanned Hospital Admissions in Heart Failure Patients: A Cohort Study**

Ofra Kalter-Leibovici^1, 2*^, Havi Murad^1^, Arnona Ziv^1^, Tomer Keidan^3^, Alon Orion^4^, Yoav Afel^5^, Harel Gilutz^6^, Dov Freimark^5^, Rachel Klibansky-Marom^1^, Laurence Freedman^1^, Haim Silber^7^.

Supplementary Information 2

**Table 1: Additional univariate comparisons of baseline characteristics by patient unplanned hospital admission profile**

|  | Patient subgroups | | | | | |  |  |
| --- | --- | --- | --- | --- | --- | --- | --- | --- |
| P* | Cluster 5  N=56 | Cluster 4  N=115 | Cluster 3  N=49 | Cluster 2  N=528 | Cluster 1  N=307 | No admission  N=305 | All patients  N=1,360 | Baseline characteristics |
| 0.38 | 46 (82.1) | 93 (80.9) | 45 (91.8) | 435 (82.4) | 255 (83.1) | 263 (86.2) | 1137 (83.6) | Treatment with angiotensin-converting enzyme inhibitors or angiotensin receptor blockers, vs. no treatment |

**Table 1: Additional univariate comparisons of baseline characteristics by patient unplanned hospital admission profile (cont.)**

|  | Patient subgroups | | | | | | | | | | |  |  |
| --- | --- | --- | --- | --- | --- | --- | --- | --- | --- | --- | --- | --- | --- |
| P* | Cluster 5  N=56 | Cluster 4  N=115 | | Cluster 3  N=49 | | Cluster 2  N=528 | | Cluster 1  N=307 | | No admission  N=305 | | All patients  N=1,360 | Baseline characteristics |
| 0.1003 | 44 (78.6) | 91 (79.1) | | 39 (79.6) | | 385 (72.9) | | 232 (75.6) | | 249 (81.6) | | 1040 (76.5) | Treatment with beta-adrenergic blockers, vs. no treatment (%) |
| 0.49 | 24 (42.9) | 46 (40.0) | | 15 (30.6) | | 189 (35.8) | | 119 (38.8) | | 126 (41.3) | | 519 (38.2) | Treatment with mineralocorticoid receptor antagonists, vs. no treatment (%) |
| 0.99 | 8 (14.3) | | 21 (18.3) | | 9 (18.4) | | 87 (16.5) | | 51 (16.6) | | 52 (17.0) | 228 (16.8) | Implanted pacemaker, N (%) |
