# Supplementary material for: Causes and predictors of recurrent unplanned hospital admissions in heart failure patients: a cohort study
Source: Intern Emerg Med. 2024 Aug 18;19(8):2213–21. doi: 10.1007/s11739-024-03740-2 (PMC11582252; doi:10.1007/s11739-024-03740-2)
Supplement: Supplementary file 3 — Supplementary file3 (DOCX 28 KB) [file 11739_2024_3740_MOESM3_ESM.docx]

**Causes and Predictors of Recurrent Unplanned Hospital Admissions in Heart Failure Patients: A Cohort Study**

Ofra Kalter-Leibovici^1, 2*^, Havi Murad^1^, Arnona Ziv^1^, Tomer Keidan^3^, Alon Orion^4^, Yoav Afel^5^, Harel Gilutz^6^, Dov Freimark^5^, Rachel Klibansky-Marom^1^, Laurence Freedman^1^, Haim Silber^7^.

Supplementary Information 3

**Supplementary Statistical Information: The assignment algorithm and its evaluation and optimization**

Algorithm for assigning patients to their subgroup

We first calculated the estimated probability to belong to each of the subgroups: *j*=0,1,2,3,4,5 ($P_{ij}$) for each subject *i*, from the multinomial model. In this notation, subgroup *j*=0 comprises patients who have no unplanned hospital admissions on follow-up, whereas subgroups *j*=1, 2, 3, 4 and 5 correspond to the 5 cluster subgroups described in the main text. We then used these estimated probabilities to define a series of algorithms for assigning patients to their subgroup. Each algorithm was constructed as follows.

Step 1: The subgroups were first ordered according to the intensity of treatment management required, from the most intense to the least intense, as follows:

1) Subgroups 4 and 5 combined; 2) Subgroup 3; 3) Subgroup 1; 4) Subgroup 2; 5) Subgroup 0.

Step 2: The assignment algorithm was defined in a hierarchical manner starting with the subgroup with highest intensity of treatment management and proceeding in decreasing order of intensity, as follows.

If $P_{i4}+P_{i5}\geq\pi_{45}$, assign patient to subgroup 4/5; otherwise, if $P_{i3}\geq\pi_{3}$, assign patient to subgroup 3; otherwise, if $P_{i1}\geq\pi_{1}$, assign patient to subgroup 1; otherwise, if $P_{i2}\geq\pi_{2}$, assign patient to subgroup 2; otherwise, assign patient to subgroup 0.

In the above, each algorithm used a different set of cut-off values ($\pi_{45}$, $\pi_{3}, \pi_{1}$ and $\pi_{2})$. After preliminary inspection, the following values were considered. For $\pi_{45}$: 0.16 - 0.24 in steps of 0.02. For the other cut-off values ($\pi_{3}, \pi_{1}$ and $\pi_{2})$: 0.1 - 0.5 in steps of 0.1. Thus for each cut-off there were 5 options, and algorithms corresponding to all 625 (5x5x5x5) combinations of these options were considered.

Evaluation of the assignment algorithms

We evaluated the goodness of prediction provided by each assignment algorithm using classification tables (i.e. a 5x5 cross-tabulation of the numbers of patients’ assigned to each subgroups versus their true subgroup, as shown in Table 1), in conjunction with ascribed losses corresponding to each type of misclassification. These loss values are also shown in Table 1 using color coding (red, orange, blue, green black, corresponding to loss values of 4,3,2,1 and 0 units respectively) for the different misclassifications.

The motivation for these loss values is as follows:

Patients in subgroups 4 and 5 have the worst prognosis and a high burden of unplanned hospital admissions, mainly due to heart failure exacerbation. These patients should be best treated intensively by a multidisciplinary team (e.g. cardiologist, nurse educator). Thus, erroneous classification of these patients to lower intensity care setting received a high (4) or intermediate (3) loss value.

Patients in subgroup 3 have somewhat better prognosis but a high hospital admission rate, mainly due to acute events of ischemic heart disease and arrhythmia. These patients should be followed frequently by a cardiologist, who will make timely decisions on the appropriate management of these conditions. Thus, erroneous classification of these patients to lower intensity care setting received a high (4) or intermediate (3) loss value. On the other hand, if erroneously assigned to group 4/5, these patients will be followed frequently by a cardiologist and eventually will receive an appropriate care. Thus, the associated loss value is low (2).

Patients in subgroup 1 have poor prognosis and moderate hospital admission rate, mainly due to decompensated heart failure. These patients should be managed jointly by their primary physician and a cardiologist. Thus, erroneous classification of these patients to lower-intensity care setting received an intermediate (3) or low (2) loss value. Misclassification to higher intensity care received a loss value corresponding to monetary, but not clinical loss (1).

Patients in subgroup 2 have significant mortality. They also have significant hospital admission rate, but for multiple causes, without any dominant cause(s) that may guide specific targeted intervention. Thus, erroneous classification of these patients to low-intensity care setting received a low loss (2).

Finally, erroneous assignment of patients in subgroups 0 and 2 to higher intensity care plan does not have clinical ramifications, so the loss value corresponded to a monetary loss (1).

**SI Table-1. Classification table corresponding to an assignment algorithm.**

Cell entries are the numbers of patients with the corresponding assigned and true subgroups. Colors represent severity of errors in classification. Red (high clinical penalty: loss = 4); Orange (intermediate clinical penalty; loss= 3); Blue (low clinical penalty: loss =2); Green (financial penalty; loss = 1); Black (No penalty; loss = 0).

| **Observed patient subgroup** | | | | |  |  |
| --- | --- | --- | --- | --- | --- | --- |
| 4/5 | 3 | 2 | 1 | 0* |  | **Assigned patient subgroup** |
| N04 | N03 | N02 | N01 | N00 | 0* |  |
| N14 | N13 | N12 | N11 | N10 | 1 |  |
| N24 | N23 | N22 | N21 | N20 | 2 |  |
| N34 | N33 | N32 | N31 | N30 | 3 |  |
| N44 | N43 | N42 | N41 | N40 | 4/5 |  |

* Subgroup 0 include patients who did not experience an unplanned hospital admission during follow-up.

After fixing these loss values, we calculated the total loss for each algorithm from the following formula:

Total loss = **4***(N03+N23+N04+N24) + **3***(N01+N13+N14) + **2***(N21+N02+N43) + (N10+N30+N40+N31+N41+N32+N42)

The assignment algorithm, among the 625 considered, that yielded the lowest total loss was considered the optimal one. The classification table for the optimal assignment is presented in Statistical Appendix Table-2.

**SI Table-2. Classification table for the assignment algorithm with the smallest total loss**

| N (column %) | Observed patient subgroup | | | | | | |
| --- | --- | --- | --- | --- | --- | --- | --- |
| Predicted patient subgroup | Patient subgroup | 0* | 1 | 2 | 3 | 4/5 | Total |
|  | 0* | 13 (4.3) | 17 (5.5) | 22 (4.2) | 1 (2.0) | 6 (3.5) | 59 |
|  | 1 | 223 (73.1) | 215 (70.0) | 364 (68.9) | 24 (49.0) | 91 (53.2) | 917 |
|  | 2 | 33 (10.8) | 6 (2.0) | 25 (4.7) | 1 (2.0) | 3 (1.8) | 68 |
|  | 3 | 5 (1.6) | 8 (2.6) | 18 (3.4) | 9 (18.4) | 4 (2.3) | 44 |
|  | 4/5 | 31 (10.2) | 61 (19.9) | 99 (18.8) | 14 (28.6) | 67 (39.2) | 272 |
|  | Total | 305 | 307 | 528 | 49 | 171 | 1,360 |

* Subgroup 0 include patients who did not experience an unplanned hospital admission during follow-up.
